# Increased cerebral lactate levels in adults with autism spectrum disorders compared to non-autistic controls: a magnetic resonance spectroscopy study

**DOI:** 10.1186/s13229-023-00577-y

**Published:** 2023-11-17

**Authors:** Simon Maier, Kathrin Nickel, Thomas Lange, Georg Oeltzschner, Michael Dacko, Dominique Endres, Kimon Runge, Anke Schumann, Katharina Domschke, Michalis Rousos, Ludger Tebartz van Elst

**Affiliations:** 1 Department of Psychiatry and Psychotherapy, Medical Center – University of Freiburg, Faculty of Medicine, University of Freiburg, Hauptstraße 5, 79104 Freiburg, Germany; 2 Medical Physics, Department of Radiology, Medical Center – University of Freiburg, Faculty of Medicine, University of Freiburg, Freiburg, Germany; 3 Russell H. Morgan Department of Radiology and Radiological Science, The Johns Hopkins University School of Medicine, Baltimore, MD USA; 4 F. M. Kirby Research Center for Functional Brain Imaging, Kennedy Krieger Institute, Baltimore, MD USA; 5 Department of General Paediatrics, Adolescent Medicine and Neonatology, Medical Center – University of Freiburg, Faculty of Medicine, University of Freiburg, Freiburg, Germany

**Keywords:** Autism spectrum disorder, Magnetic resonance spectroscopy, Lactate, Posterior cingulate cortex, Mitochondria, Mitochondrial dysfunction

## Abstract

**Introduction:**

Autism spectrum disorder (ASD) encompasses a heterogeneous group with varied phenotypes and etiologies. Identifying pathogenic subgroups could facilitate targeted treatments. One promising avenue is investigating energy metabolism, as mitochondrial dysfunction has been implicated in a subgroup of ASD. Lactate, an indicator of energy metabolic anomalies, may serve as a potential biomarker for this subgroup. This study aimed to examine cerebral lactate (Lac+) levels in high-functioning adults with ASD, hypothesizing elevated mean Lac+ concentrations in contrast to neurotypical controls (NTCs).

**Materials and methods:**

Magnetic resonance spectroscopy (MRS) was used to study cerebral Lac+ in 71 adults with ASD and NTC, focusing on the posterior cingulate cortex (PCC). After quality control, 64 ASD and 58 NTC participants remained. Lac+ levels two standard deviations above the mean of the control group were considered elevated.

**Results:**

Mean PCC Lac+ levels were significantly higher in the ASD group than in the NTC group (*p* = 0.028; Cohen’s *d* = 0.404), and 9.4% of the ASD group had elevated levels as compared to 0% of the NTCs (*p* = 0.029). No significant correlation was found between blood serum lactate levels and MRS-derived Lac+ levels.

**Limitations:**

A cautious interpretation of our results is warranted due to a *p* value of 0.028. In addition, a higher than anticipated proportion of data sets had to be excluded due to poor spectral quality.

**Conclusion:**

This study confirms the presence of elevated cerebral Lac+ levels in a subgroup of adults with ASD, suggesting the potential of lactate as a biomarker for mitochondrial dysfunction in a subgroup of ASD. The lower-than-expected prevalence (20% was expected) and moderate increase require further investigation to elucidate the underlying mechanisms and relationships with mitochondrial function.

**Supplementary Information:**

The online version contains supplementary material available at 10.1186/s13229-023-00577-y.

## Background

Autism spectrum disorders (ASDs) encompass a wide range of phenotypic expressions and are rooted in complex etiologies involving an interplay of environmental and genetic factors [1–4]. Diagnosis is currently based solely on behavioral criteria, and despite investigations of various potential diagnostic biomarkers, including metabolic markers, none have emerged as universally applicable or sufficiently specific [5–8]. This complexity highlights the need to explore subgroups within ASD that are characterized by specific genetic, metabolic, and behavioral markers. Markers of energy metabolism, including lactate, are emerging as important pathogenetic midstream markers, potentially indicating a subgroup with mitochondrial dysfunction [9–11].

Lactate is produced during anaerobic metabolism and serves as a critical metabolic intermediary in several organs, particularly the brain, where it is thought to facilitate energy transfer between astrocytes and neurons [12, 13]. Elevated lactate levels are common in mitochondrial disease (MD), but it is important to note that they are not specific to MD, and normal lactate levels do not definitively rule out MD [14–17].

MD can result from factors such as genetic variation [18], oxidative stress [19], environmental toxins [20], and certain drugs such as valproic acid [21]. Conversely, mitochondrial dysfunction refers to conditions with suboptimal mitochondrial function that do not reach the severity commonly seen in MD.

The link between energy metabolism and ASD was first described by Coleman and Blass in 1985 [22]. They identified lactic acidosis in several autistic individuals, suggesting the existence of a subgroup with carbohydrate metabolism abnormalities that affect mitochondrial function. Subsequent research using blood sample analysis has consistently observed elevated lactate and pyruvate levels in ASD [23–30]. A meta-analysis of 68 studies by Rossignol and Frye [31] found that over 30% of children with ASD have elevated lactate levels. The same meta-analysis highlighted a significantly higher prevalence of MD in individuals with ASD (approximately 5%) compared to the general population (0.02%) [32].

Compared to research on peripheral lactate levels in ASD, there is a limited number of studies investigating brain lactate concentrations using proton magnetic resonance spectroscopy (^1^H-MRS). Most previous ^1^H-MRS investigations in ASD have focused primarily on metabolites other than lactate, specifically glutamate [33] or gamma-aminobutyric acid (GABA) [34]. Almost all previous studies of brain lactate in ASD have used 1.5 T scanners and found no significant differences between individuals with ASD and neurotypical controls (NTC) [35–40]. Only one of these studies reported detecting a lactate peak in one out of 15 children with ASD and none among the control group [35]. However, a more recent study by Goh et al. [41], using proton multiplanar spectroscopic imaging on a 3 T system, which has a higher sensitivity for detecting lactate [42], analyzed several brain regions in a larger sample of 75 individuals with ASD and 96 controls ranging in age from 5 to 60 years. The study found higher rates of lactate doublets in several brain regions, including the basal ganglia, corpus callosum (CC), and cingulate structures in participants with ASD (13%) compared to controls (1%), and a significant correlation with age in the ASD group. No other MRS studies investigating lactate levels in individuals with autism could be found despite an extensive search.

Lunsing et al. [43] showed that brain tissue lactate levels measured by quantitative MRS could serve as a diagnostic marker for MD in children, possibly with higher sensitivity compared to CSF-derived measurements.

Previous studies investigating brain lactate levels using MRS have relied on 1.5 T MR systems, which are not ideal for lactate measurement [42], or magnetic resonance spectroscopic imaging (MRSI) [41], which, while capable of imaging multiple brain regions simultaneously, typically has reduced sensitivity compared to single-voxel spectroscopy (SVS). This MRSI study detected the presence of lactate peaks without quantifying them. Therefore, our study for the first time used lactate-edited single-voxel MRS with a 3 T MR system to measure lactate concentrations in participants with and without ASD. We focused on the posterior cingulate cortex (PCC) for two main reasons: This area exhibited lactate doublets in a previous study by Goh et al. [41], and our pilot tests revealed superior spectral signal quality in the PCC compared to other regions, such as the anterior cingulate cortex. Based on previous data, we hypothesized that: (1) there will be elevated cerebral lactate signals in some individuals with ASD, with approximately 20% (as reported in adults by Goh et al. [41]) displaying significantly elevated concentrations, and (2) those with elevated peripheral lactate levels will also have significantly higher MRS-measured cerebral lactate signals.

## Methods

### Participants

MRS spectra were acquired from 71 individuals with ASD and 71 NTC recruited at the Department of Psychiatry and Psychotherapy at the University Medical Center Freiburg, Germany, following the Declaration of Helsinki and approval by the University Ethics Committee (Approval ID: 268/17). Participants gave written informed consent.

Participants with ASD, including Asperger’s Syndrome as defined by the Diagnostic and Statistical Manual of Mental Disorders (DSM-5; 299.0) and International Classification of Diseases (ICD-10; F84.5), respectively, were enrolled, excluding those with known secondary genetic forms of ASD. The diagnoses were established by experienced senior psychiatrists with expertise in ASD according to the ICD-10 (F84.5) as well as DSM-5 criteria (299.0). In addition, the following questionnaires were administered: Autism Spectrum Quotient (AQ) [44], Empathy Quotient (EQ) [45], and Social Responsiveness Scale 2 (SRS-2) [46, 47]. In cases of uncertainty, Autism Diagnostic Interview Revised (ADI-R) [48, 49] and the Autism Diagnostic Observation Schedule (ADOS) [50] were additionally utilized. The NTC group was recruited via advertisements.

Participants underwent a battery of diagnostic tests, including the Structured Clinical Interview (SCID I and II) [51, 52], Autism Spectrum Quotient (AQ) [44], Empathy Quotient (EQ) [45], Social Responsiveness Scale 2 (SRS-2) [46, 47] for ASD assessment; Beck Depression Inventory (BDI-II) [53, 54] for depressive symptoms; Wender Utah Rating Scale (WURS-k) [55, 56] for attention-deficit/hyperactivity disorder (ADHD) symptoms in childhood; Multiple Choice Vocabulary Test (MWT-B) [57, 58] for crystallized intelligence (IQ); Symptom-Checklist-90® (SCL-90-R) [59] for physical and psychiatric symptoms; and State Anxiety Inventory (STAI) [60, 61] for current symptoms of anxiety between blood sampling and before scanning.

Exclusion criteria included MRI contraindications (e.g., pregnancy, metal implants, claustrophobia), metabolic conditions (e.g., diabetes mellitus), obesity (BMI > 30 kg/m^2^), neurological disorders such as seizures, and regular benzodiazepine use. Treated hypothyroidism did not result in study exclusion. For ASD participants, bipolar disorder, psychotic symptoms, and substance abuse were additional exclusion criteria. The NTC group consisted of participants without psychiatric disorders as assessed by the SCID, SCL-90-R, SRS-2, and WURS-k.

The day before data collection, participants were asked to refrain from physical exercise.

### Data acquisition

Magnetic resonance imaging data were acquired at the Radiology Department of the University Medical Center Freiburg, Germany, using a Magnetom Prisma 3 T system (Siemens Healthineers, Germany) with a 20-channel head coil. A T1-weighted magnetization prepared rapid gradient echo (MPRAGE) protocol provided an anatomical 3D dataset (sagittal orientation, TR = 2000 ms, TE = 4.11 ms, FOV = 256 × 256 × 160 mm^3^, flip angle = 12°, voxel size = 1 × 1 × 1 mm^3^) for MRS planning and tissue segmentation.

In the spectroscopic measurements, a MEGA-semiLaser sequence [62] (TR = 1650 ms, TE = 142 ms, flip angle = 90°, 192 averages, voxel center frequency = 2.0 ppm) was used for lactate editing. The editing pulse (full width half maximum (FWHM) bandwidth = 60 Hz) was applied at 4.1 ppm in the edit-ON shots and 5.3 ppm in the edit-OFF shots. For absolute water quantification using the water reference method [63], the same protocol was run without water suppression, with a voxel center frequency of 4.7 ppm and 16 spectral averages.

**Fig. 1:**
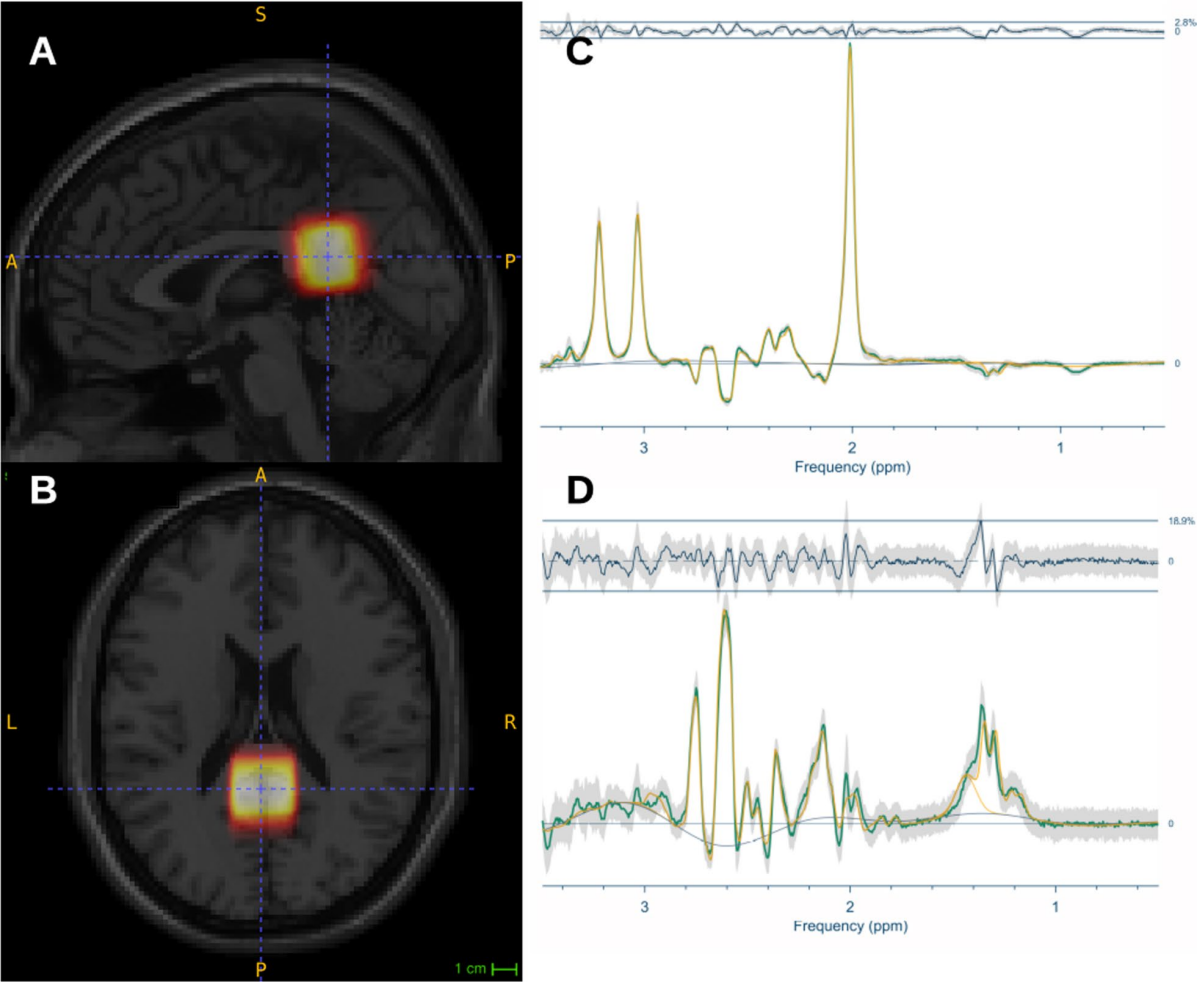
MRS voxel Placement in the Posterior Cingulate Cortex and Aggregate OFF and Difference Spectra. The figure displays the superimposed MRS voxel placements for all participants within the posterior cingulate cortex, as shown in the sagittal (**A**) and transversal (**B**) views. The average spectra are represented by the green line, with the gray shading indicating standard deviation, and the yellow line depicting the model fit for the editing-off (**C**) and difference editing spectra (**D**)

SVS MRS voxels (25 × 25 × 25 mm^3^) were positioned over the splenium, specifically near the tentorium cerebelli in the occipital region of the CC to maximize the signal-to-noise ratio and capture the PCC while minimizing cerebrospinal fluid inclusion (cf. Fig. 1 and Additional file 1: Information 1). Detailed parameters are shown in Additional file 1: Table S1.

Heart rate and respiration were monitored during the session to detect acute anxiety or hyperventilation, as they can elevate brain lactate levels [64–68].

Of the 71 collected MRS spectra, three from the ASD group and two from the NTC group were unanalyzable due to file corruption. We qualitatively assessed all spectra for artifacts and fit quality. Datasets from four ASD subjects and eleven NTC subjects had spectral artifacts, primarily spurious echo artifacts [69] affecting peaks down-field of 3 ppm, resulting in poor fitting. Using data exclusion thresholds for tCr (FWHM > 8 Hz) [70], the final sample included 64 ASD and 58 NTC subjects after exclusion.

Our MRS parameter reporting follows the Minimum Reporting Standards as per in vivo MRS experts’ consensus recommendations [71].

Information about blood collection, processing, and analysis is published elsewhere [72].

### Data processing

Data analysis was performed using Osprey (v2.5.0) [73], an open-source MRS analysis toolbox in MATLAB (R2022a). We used Osprey’s Linear Combination Model (LCM) algorithm to model the lactate-edited difference spectra and the edit-OFF spectra, using a basis set of twenty metabolites generated by density matrix simulations based on the FID-A toolbox [70]. The basis set included the twenty metabolites Ala (alanine), Asp (aspartate), bHB (beta-hydroxybutyrate), tCr (total creatine), GABA, GPC (glycerophosphocholine), GSH (glutathione), Gln (glutamine), Glu (glutamate), H_2_O (water), mI (myo-inositol), Lac (lactate), NAA (*N*-acetylaspartate), NAAG (*N*-acetylaspartylglutamate), PCh (phosphocholine), PE (phosphoethanolamine), sI (scyllo-inositol), Tau (taurine), Thr (threonine). In the lactate-edited difference spectrum, we added two Gaussian components (MM12 and MM14) to account for co-edited macromolecular (MM) signals, as described in our previous investigation of the acquisition technique [62]. A spline baseline with 0.55 ppm knot spacing accounted for background fluctuations. Fitting was performed in the spectral domain range (0.5–3.55 ppm). Average spectra are shown in Fig. 1 and Additional file 1: Fig. S1.

As lactate and threonine have resonances at similar spectral frequencies (~ 1.31 ppm), their signals strongly overlap, complicating distinct identification. Due to this overlap, our primary outcome was the sum of lactate and Thr, referred to as Lac+.

### Statistical analysis

#### Test for normality

We initially used the Shapiro–Wilk test to verify the normal distribution of five Lac+ estimates from Osprey. These were: (1) the unadjusted Lac+ metabolite amplitude; (2) the raw water-scaled Lac+ concentration, i.e., the Lac+ metabolite concentration divided by the water amplitude; (3) the water-scaled and CSF-corrected Lac+  concentration; (4) the water-scaled, CSF-corrected, and tissue-relaxations-corrected Lac+ concentration; and (5) the Lac+/tCR ratio. The data were normally distributed for all estimates and for both groups (ASD and NTC).

#### Fractional volume effects

Informed by theories such as the lactate-shuffle theory, suggesting differences in lactate concentrations across gray matter (GM), white matter (WM), and cerebrospinal fluid (CSF), we conducted an empirical investigation. The aim was to determine which of the five lactate estimates (cf. 2.4. Test for Normality) best minimized these fractional volume effect. Since non-collinearity of independent variables is a fundamental assumption in ANCOVA models, we addressed the inherent collinearity between the voxels’ fractional GM, WM, and CSF volumes. Refer to Additional file 1: Information 2 for the method of calculating orthogonal fractional volume components (FVC1 and FVC2) from GM, WM, and CSF, and for details on the rationale for using the raw water-scaled lactate concentration (Lac+) in further analyses. When comparing the above mentioned lactate estimates from Osprey, the raw water-scaled lactate concentration was the measure that was least biased by fractional volume effects (cf. Additional file 1: Table S3).

#### Effect of demographic and MRS quality-related covariates on Lac+

Our next goal was to identify demographic and MRS quality-related covariates that might significantly impact Lac+ concentrations. (Group differences in measures of spectral quality are shown in Additional file 1: Table S2.) We examined the effects of age, relative residual differences, FVC1, FVC2, sex, and frequency shift in a linear regression model. Since none of these covariates showed a significant effect on Lac+ concentrations, we excluded these covariates from further analyses.

#### Lac+between-group comparison

After confirming that no covariates significantly influenced Lac+ concentrations and the Lac+ data were normally distributed, we analyzed group differences with a two-sample t test. We repeated the t test excluding participants with a blood lactate concentration greater than 1.5 mmol/L. Refer to Additional file 1: Information 3 and Additional file 1: Table S3 for confirmatory analyses regarding other Lac+ estimates derived from Osprey.

In two additional ANCOVA models with the same covariates, we examined the group effect on tissue-corrected and water-scaled concentrations of NAA and tCr. We tested the assumptions for the ANCOVA models, including homogeneity of variances between groups with Levene’s test and normality of residuals with the Shapiro–Wilk test. Because tCr failed the normality test, we log-transformed tCr concentrations and reran the ANCOVA.

In addition, we investigated whether a subgroup within the ASD population had elevated cerebral Lac+ concentrations. We established a reference range based on the control group, defining the upper limit of normal Lac+ concentrations as two standard deviations above the mean. We identified ASD and NTC subjects with and without elevated Lac+ levels and compared the frequency of elevated Lac+ levels between groups using Fisher’s exact test.

#### Serum lactate between-group comparison

Assessment of serum lactate levels revealed a non-normal distribution as determined by the Shapiro–Wilk test. To address this, we employed a Tukey’s Ladder of Powers transformation to mitigate data skewness. While this transformation did lessen the skewness, the distribution remained significantly non-normal according to a subsequent Shapiro–Wilk test. Consequently, we utilized robust Yuen’s independent samples *t* test for trimmed means (WRS2 package) [74, 75] to probe group differences in the Tukey-transformed lactate levels. This analysis focused on the MRS subsample of 64 individuals with ASD and 58 without, drawn from a larger original cohort of 73 ASD and 71 NTC participants, as described in our previous publication investigating blood markers of mitochondrial function [72].

#### Correlation of Lac+ with psychometric scores

To explore the relationship between Lac+ concentrations and psychometric scores (including SRS-2, AQ, EQ, BDI-II, WURS-k, STAI, IQ) and participants’ age, we computed Spearman’s correlation coefficients for both groups and the ASD group specifically. Additionally, we computed correlation coefficients of the fractional GM, WM, and CSF volumes (fGM, fWM, and fCSF) with the psychiatric scores and age. Adjustments for multiple comparisons were not made at this stage as our focus was on exploring data relationships, not confirmatory claims. These correlations were visualized using a correlation matrix in R's corrplot package.

#### Correlation of Lac+ with serum lactate

We also examined the link between brain Lac+ concentrations in the PCC and serum lactate levels using Spearman’s correlation coefficients for both the combined and ASD groups.

#### Influence of medication on Lac + concentration

To assess the influence of medication on PCC Lac+ concentrations, we used an ANCOVA model with Lac+ concentrations as the dependent variable, group as the independent variable, and a variety of medications as separate covariates. The medications considered included antipsychotics, atypical antipsychotics, serotonin–norepinephrine reuptake inhibitors (SNRIs) or selective serotonin reuptake inhibitors (SSRIs), other antidepressants, lithium, stimulants, anticonvulsants (lamotrigine), benzodiazepines (“standby medication”), and oral contraceptives.

#### Influence of day time on Lac+concentrations

To account for potential variations in lactate levels due to time of day, we ran two separate models: In a four-time-period model, we divided the day into four periods: morning (7–10 am), noon (11 am–1 pm), afternoon (2–4 pm), and evening (5–8 pm). An ANCOVA model was run with Lac+ concentrations as the dependent variable, group as the independent variable, and time of day categorization as a covariate. In a two-time-period model, we performed the same ANCOVA analysis but divided the day into two parts: before noon (7:00 am–12:00 pm) and after noon (1:00 pm–8:00 pm).

In addition, we measured one of the authors on 2 days in the morning and evening. Details are provided in Additional file 1: Information 4.

## Results

### Demography, psychometry, physiology, and medication

The two groups did not show significant differences in age, gender, and IQ, as shown in Table 1. However, they differed in autistic symptoms (AQ, EQ, SRS-2), ADHD symptoms in childhood (WURS-k), and depressive symptoms (BDI-II). In addition, the ASD group showed increased anxiety symptoms (STAI) before entering the scanner compared to the NTC group. These findings and the number of patients taking psychiatric medication are summarized in Table 1. The psychotropic drugs our patients receive do not pose an increased risk of lactic acidosis when taken at the recommended dosage. No patient took benzodiazepines on the days before the measurement or on the day of the measurement. None of the participants showed signs of a panic attack during scanning, such as hyperventilation or tachycardia, and none reported acute anxiety or panic in the debriefing.

**Table 1:** Demographic, Psychometric, and Medication Data for ASD and NTC Groups

Characteristic	ASD, *N* = 64^1^	NTC, *N* = 58^a^	*p* value^b^
Sex			0.3
Female	22 (34%)	25 (43%)	
Male	42 (66%)	33 (57%)	
Age	36 ± 11.8	32 ± 9.6	0.2
BDI-II	15.5 ± 13.0	4.2 ± 4.8	< 0.001
AQ	35.7 ± 8.1	12.1 ± 5.7	< 0.001
EQ	21.0 ± 10.3	47.1 ± 11.0	< 0.001
SRS-2	108.4 ± 28.7	34.3 ± 14.5	< 0.001
WURS-k	26.9 ± 16.0	13.6 ± 10.5	< 0.001
IQ	115 ± 14.5	113 ± 12.6	0.6
STAI	23.7 ± 10.3	11.2 ± 6.0	< 0.001
Typical antipsychotics	2 (3.1%)	0 (0%)	0.5
Atypical antipsychotics	6 (9.4%)	0 (0%)	0.029
SNRI/SSRI	15 (23%)	0 (0%)	< 0.001
Other antidepressants	15 (23%)	0 (0%)	< 0.001
Lithium	2 (3.1%)	0 (0%)	0.5
Stimulants	4 (6.3%)	0 (0%)	0.12
Anticonvulsant	1 (1.6%)	0 (0%)	> 0.9
Benzodiazepines (standby)	2 (3.1%)	0 (0%)	0.5
Oral contraceptives	2 (3.1%)	4 (6.9%)	0.4

*SRS-2* Social Responsiveness Scale, *AQ* Autism Spectrum Quotient, *EQ* Empathy Quotient, *BDI-II* Beck Depression Inventory, *WURS-k* Wender Utah Rating Scale, *STAI* State Anxiety Inventory, *SNRI* serotonin–norepinephrine reuptake inhibitor, *SSRI* selective serotonin reuptake inhibitor

Significance levels: *p < 0.05, **p < 0.01, ***p < 0.001

^a^*n* (%); Mean ± SD

^b^Fisher’s exact test, two-sample *t* test, and Wilcoxon rank sum test

### Differences in PCC metabolite concentrations between ASD and NTC

**Fig. 2:**
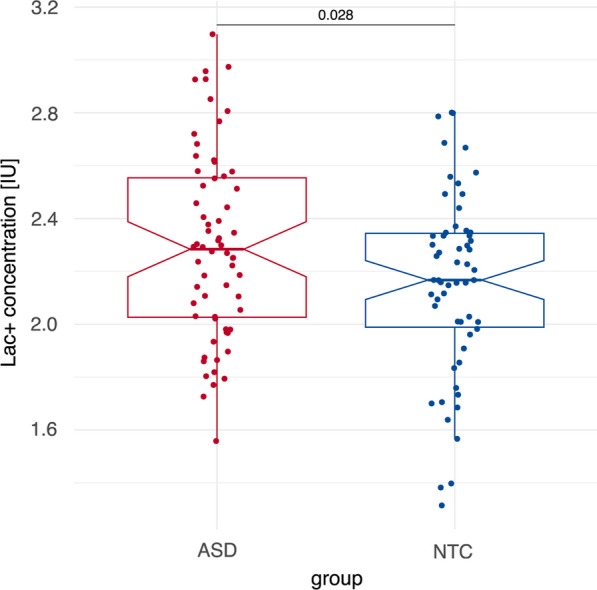
Comparison of Lac+ Concentration in the Posterior Cingulate Cortex between ASD and NTC Groups via Boxplot. This figure illustrates the significant difference (*p* = 0.028) in the mean Lac+ concentration between the ASD and NTC groups in the posterior cingulate cortex. Notches indicate the standard deviation

There was a significant difference in Lac + concentrations between the ASD and NTC groups, as shown by the two-sample t test analysis (*t*(119.25) = 2.23, *p* = 0.028, Cohen’s *d* = 0.404; cf. Fig. 2). After excluding subjects (one NTC and one ASD participant) with elevated serum lactate concentrations (> 1.5 mmol/L), the *t* test remained significant (*t*(117) = 2.08, *p* = 0.0399, Cohen’s *d* = 0.380). No significant group effect was found in exploratory analyses of total NAA (tNAA = NAA + NAAG) (*F*(1,114) = 1.546, *p* = 0.216) and log-transformed tCr (*F*(1,114) = 0.056, *p* = 0.813). Detailed information on additional lactate estimates and exploratory analyses of tNAA and log-transformed tCr concentrations can be found in Additional file 1: Table S3 and Additional file 1: Information 3.

Using a reference range based on the control group—where the upper limit of normal Lac+ concentrations was defined as two standard deviations above the mean—we identified six individuals within the ASD group who had elevated Lac + concentrations (9.4%), compared to none in the NTC group. This difference in the frequency of elevated Lac+ concentrations was significant (*p* = 0.029), with all six ASD individuals having Lac+ concentrations between two and three standard deviations above the mean for the NTC group.

### Differences in serum lactate concentrations between ASD and NTC

Group comparisons of blood serum lactate levels in the current sample—a subset derived from a previously published larger sample [72]—revealed significantly lower serum lactate concentrations in the ASD group compared to the NTC group (*t*(NA) = -2.25, *p* value = 0.029).

### Correlation with psychometric scores

**Fig. 3:**
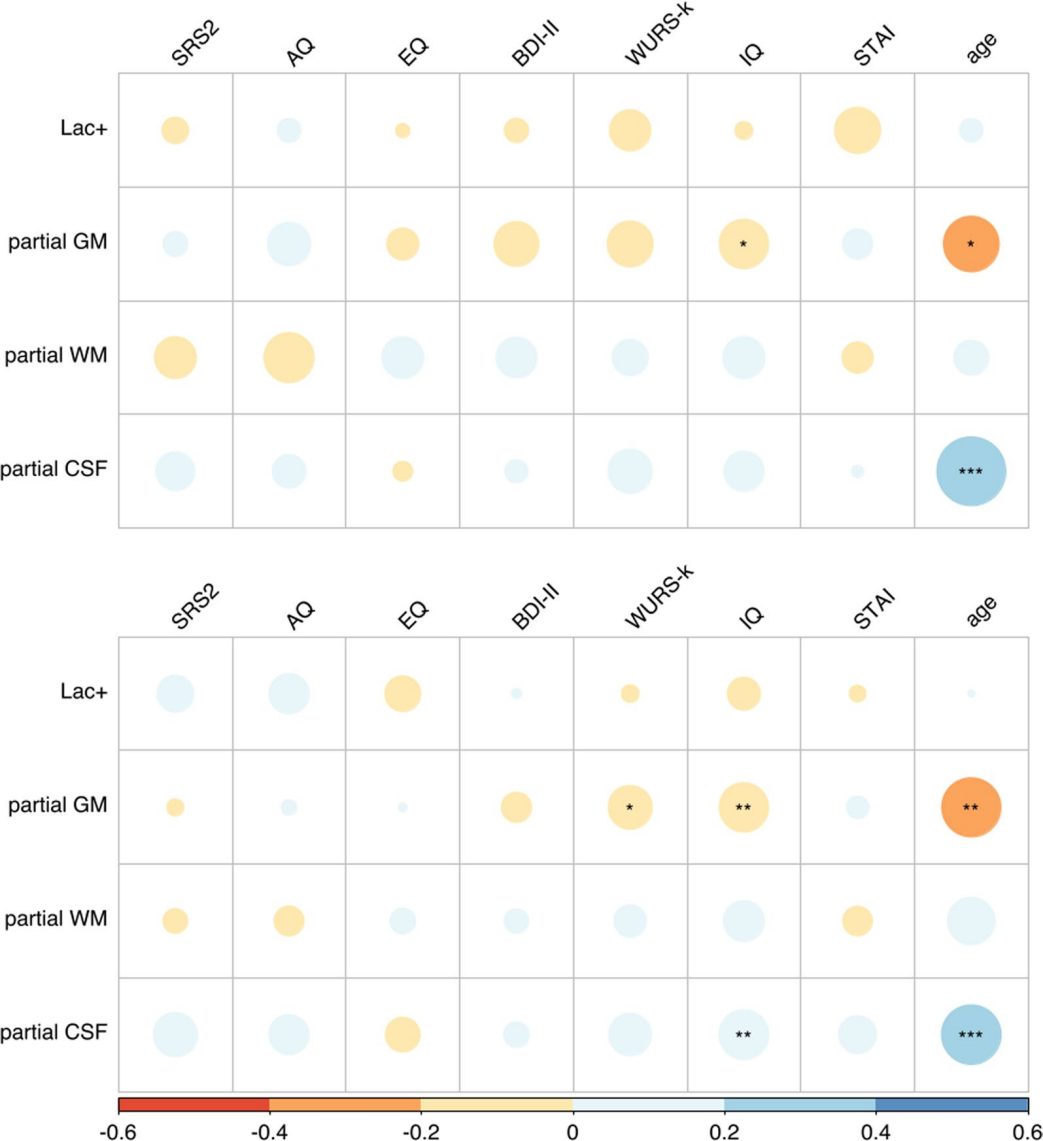
Correlation Matrix of Psychometric Scores and Age with Lac+ Concentration and fractional Voxel Volumes. This matrix shows the correlations among psychometric scores and age with Lac+ concentration and GM/WM/CSF voxel volume fractions in the posterior cingulate cortex for the ASD group (upper graph) and both groups combined (lower graph). Circle size and color indicate the strength of the correlation (“R”). Notations: SRS-2 = Social Responsiveness Scale, AQ = Autism Spectrum Quotient, EQ = Empathy Quotient, BDI-II = Beck Depression Inventory, WURS-k = Wender Utah Rating Scale, STAI = State Anxiety Inventory, GM = Gray Matter, WM = White Matter, CSF = Cerebrospinal Fluid. Significance levels: *p < 0.05, **p < 0.01, ***p < 0.001

In the correlation analyses performed for the ASD group and for both groups combined, no significant correlation was found between Lac+ concentrations and psychometric scores or age of the participants (cf. Fig. 3). Only fractional volumes showed correlations with these scores. Specifically, there was a significant negative correlation between fGM and ADHD symptoms (as measured by WURS-k scores), age, and IQ across both groups (see Fig. 3). In addition, there was a significant positive correlation between fCSF, age, and IQ. When the analysis was restricted to the ASD group only, only the negative correlation between fGM and age and IQ and the positive correlation between fCSF and age remained significant (cf. Fig. 3).

### Correlation with serum lactate

**Fig. 4:**
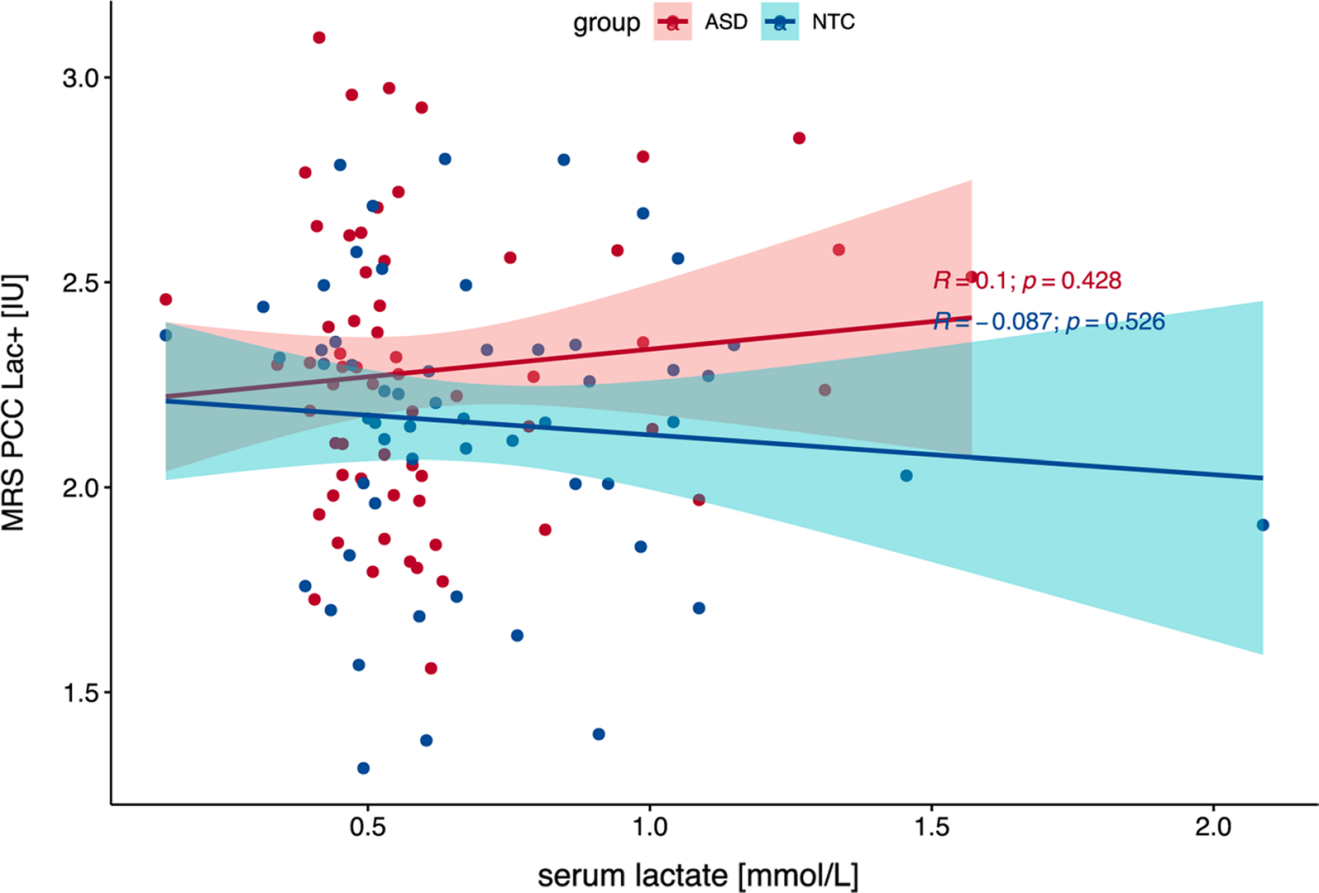
Scatterplot of Serum and Cerebral Lactate Levels in Posterior Cingulate Cortex. The scatterplot shows serum lactate concentration (*x* axis) against cerebral Lac+ concentration in the posterior cingulate cortex for the ASD group (red) and NTC group (blue). Regression lines with confidence intervals demonstrate no significant relationship between serum and cerebral lactate levels

Our analysis indicated no significant correlation between serum lactate and Lac+ concentrations in the PCC for either the ASD group or the combined groups (cf. Fig. 4). We rerun the analysis excluding one NTC and one ASD participant with increased serum lactate levels (> 1.5 mmol/L). The correlation remained insignificant (*R* = − 0.04, *p* = 0.792). Details of the group differences in serum lactate levels between the ASD and NTC groups (*T* = − 2.58, *p* = 0.012) are reported elsewhere [72].

### Influence of medication on Lac+ concentration

Analysis with an ANCOVA model including all medications as covariates showed that the group effect identified in the t test remained significant (*F*(1,111) = 4.556; *p* = 0.035). Of the medications considered, only anticonvulsants showed a significant effect on Lac+ concentrations (*F*(1,111) = 4.921; *p* = 0.029). It is important to note that this effect was attributed to only one individual with ASD who was taking anticonvulsants (lamotrigine) as an off-label medication.

### Influence of day time on Lac+ concentration

Analyses with the ANCOVA models of the four-time-period model showed a significant group effect (*F*(1,117) = 5.186; *p* = 0.025) and no significant effect for day time (*F*(3,117) = 0.344; *p* = 0.794). The two-time-period model also showed a significant group effect (*F*(1,117) = 5.198; *p* = 0.024) but no significant effect for day time (*F*(3,117) = 0.506; *p* = 0.478).

## Discussion

In this MRS study, we were able to confirm the hypothesis that cerebral Lac+ levels in the PCC are elevated in adults with high-functioning ASD compared to NTC. However, only six individuals (9.4%) from the ASD group had elevated Lac+ levels (none from the NTC group), lower than the originally hypothesized 20%. There was no significant correlation between blood serum lactate levels and MRS-derived Lac+ levels in the PCC, and those with elevated cerebral Lac+ in the ASD group had normal serum lactate levels. In addition, there was no significant correlation between Lac+ concentrations and psychometric scores, including autism and ADHD symptom severity, depression, and IQ.

The finding of elevated cerebral Lac+ in the PCC is consistent with previous research by Goh et al. [41], who observed a higher prevalence of lactate doublets in several brain regions that included the CC and cingulate structures and coincided with or were in close proximity to the location of the PCC voxel examined in our study. This supports the literature that has relatively consistently reported elevated serum lactate levels in ASD [23–28], but contrasts with null results from previous MRS studies on 1.5 T MR systems [35–40], underscoring the importance of selecting the appropriate MRI methodology. In the MRS subsample analyzed in the current study, serum lactate levels were consistent with previously reported findings in the larger sample, with the ASD group having significantly lower lactate concentrations compared to the NTC group [72]. Notably, none of the individuals with ASD had abnormally high serum lactate levels or clinical signs suggesting the need for further investigation of mitochondrial dysfunction [72]. This could point to a possible compartmentalization of metabolic dysregulation, where the brain, but not the blood, exhibits increased lactate levels indicative of potential metabolic dysfunction specific to ASD. The six individuals with ASD with elevated cerebral Lac+ showed moderate elevations in lactate concentration (between two and three standard deviations). The effect size of the difference in cerebral Lac+ concentration between groups was small.

The question remains whether, for a subset of individuals with ASD, mitochondrial dysfunction could be a potential pathomechanistic marker. In our study of high-functioning adults with ASD, there was no evidence of mitochondrial dysfunction based on blood analysis of energy metabolism markers and cerebral lactate concentrations [72]. It is important to note that not all mitochondrial disorders result in elevated lactate levels [14–17] and the vast majority of mitochondrial diseases lack sufficient biomarkers. Furthermore, the estimated prevalence of over 30% of children with ASD having elevated serum lactate levels [31] was not reflected in our adult sample, where only one ASD and one NTC had a lactate concentration above 1.5 mmol/L. The 9.4% of ASD participants in our sample with elevated cerebral lactate is more consistent with the 13–20% prevalence of lactate doublets in individuals with ASD reported by Goh et al. [41].

In our study, we found no between-group differences in tNAA and tCR, which is in contrast to some pediatric ASD studies that have reported lower NAA levels [76, 77]. Since NAA is predominantly synthesized in the mitochondria of neurons and in oligodendrocytes, one might expect that elevated lactate, as an indicator of mitochondrial dysfunction, would correspond to lower NAA levels [78, 79]. However, other adult studies have also reported no differences in NAA [80]. Decreased NAA levels in children may indicate a reduction in neuronal density during developmental stages [76, 80]. However, elevated lactate levels do not imply a reduction in neuronal density.

While elevated lactate is typically associated with mitochondrial dysfunction, other factors such as stress, [81], physical activity [82, 83], and diet may also contribute [84]. We controlled for physical activity and found a significant decrease in blood lactate rather than an increase in the ASD group. In addition, Maddock et al. reported only a relatively small increase in cerebral lactate after rigorous exercise [83]. While none of the subjects showed signs of an acute stress response in the form of hyperventilation or tachycardia during the measurement, which would also affect blood lactate levels [67], elevated state anxiety scores could indicate higher stress levels [81], which could affect cerebral lactate levels. A high carbohydrate and low glycemic index diet may also affect lactate levels [84], which we did not control for or assess in our study (see Limitations). The discrepancies in MRS studies assessing cerebral lactate levels may be due to methodological differences. Previous MRS studies reporting no increase in cerebral lactate levels used 1.5 T systems [35–40], and the lower field strength combined with small sample sizes in some studies [35, 37, 38] may have limited their sensitivity [85] and power to detect differences or subgroups with elevated cerebral lactate levels. The only previous study reporting a significantly higher prevalence of lactate doublets in ASD used a 3 T MR system [41]. None of these MRS studies used spectral editing techniques. Therefore, lactate quantification may be confounded by the presence of large overlapping lipid resonances [86].

In summary, previous MRS studies have generally reported no increase, a smaller increase, or a smaller proportion of individuals with ASD with elevated cerebral lactate levels [35–38, 38, 40, 40, 41] compared to serum lactate studies [31], which primarily reported higher lactate concentrations. This is in contrast to our study where only cerebral lactate levels were elevated in the ASD group. These discrepancies may be due in part to the focus of our study on high-functioning adults with ASD who do not have known genetic syndromes associated with autism and mitochondrial dysfunction, such as fragile X syndrome or the 15q11-q13 inverted duplication [87, 88]. In addition, the focus on adults may explain the lower prevalence of elevated lactate levels compared to studies using peripheral measurements [89], although the findings of Goh et al. [41], who reported a higher prevalence of lactate doublets in adults, would suggest otherwise.

This study highlights the importance of a comprehensive approach to screening for energy metabolism abnormalities such as elevated lactate levels in multiple compartments and tissues, including blood serum, cerebrospinal fluid, and brain. The study by Goh et al. [41] suggests that lactate signals may not be distributed uniformly across brain regions, and the reasons for this—whether due to localized brain activity [89] or genetic mosaicism [90–95]—warrant further investigation.

Future research could use MRS techniques with high sensitivity to quantify lactate across various brain regions and might monitor lactate concentration changes in response to stimulus-induced brain activity [89, 96]. In addition, it would be beneficial for MRS studies investigating lactate to include serum lactate measurements and other markers of mitochondrial dysfunction.

### Limitations

Although our main result reached statistical significance with a *p* value of 0.028, we acknowledge that there is ongoing debate regarding the traditional threshold for statistical significance of *p* < 0.05, with some recent suggestions advocating for a more stringent threshold of *p* < 0.005. Given this context, our findings should be interpreted cautiously and replicated in further studies to confirm their robustness.

We also acknowledge that intra-individual variation in lactate levels, which may be influenced by various factors such as time of day, physical status, carbon-rich diet, and exercise load, is a limitation of our study and warrants careful consideration in future research. Although participants were asked to refrain from physical activity the day before data collection and we did not find a significant effect of time of day in our statistical models, longer-term effects, diurnal and dietary effects cannot be ruled out.

While medication does not appear to be the main factor in the increased Lac+ levels observed in the ASD group, other non-mitochondrial factors may be contributing. In particular, the ASD group had significantly higher anxiety levels before entering the scanner, and although there were no clear signs of hyperventilation or panic, increased muscle tension due to anxiety could cause a slight increase in Lac+. However, we did not find a significant correlation between Lac+ signals and anxiety scores. Future studies may benefit from including additional measures of respiratory status, such as capnometry. In addition, our study had to exclude several participants due to severe artifacts in the MR spectra, possibly caused by head motion. Future research could monitor head motion during the MRI scan or even correct it prospectively [97]. Alternatively, improved head stabilization methods could be used, keeping in mind that the latter may increase anxiety, especially in the ASD group. Finally, it should be noted that standard MEGA editing techniques cannot distinguish lactate from Thr signals. This detection ambiguity could be resolved with low-bandwidth editing pulses, which require a real-time update of the RF carrier frequency during the MRS scan [98]. This may also decrease the amount of co-edited MM resonances and therefore reduce the variability associated with the necessary parametrization of their signals during linear-combination modeling.

### Conclusion

Elevated lactate levels may suggest mitochondrial dysfunction in some individuals with ASD. It is possible that a mild form of mitochondrial dysfunction, localized to specific areas, could cause the elevated cerebral lactate levels. Future research should thoroughly investigate the causes of these elevated lactate levels, explore whether they are related to mitochondrial dysfunction, and try to understand their distribution in different areas and tissues. It is also crucial to investigate whether these lactate elevations in ASD are due to specific causes, such as mitochondrial DNA mutations [99].

### Supplementary Information

**Additional file 1. **Supplementary information, tables and figure.

## Data Availability

Demographic, psychometric, serum blood data, MRS-derived data, as well as basis sets, and the R code for statistical analysis, are available from the corresponding author on request.

## Abbreviations

^1^H-MRS: Proton magnetic resonance spectroscopy
ADHD: Attention-deficit/hyperactivity disorder
ANCOVA: Analysis of covariance
ASDs: Autism spectrum disorders
Ala: Alanine
AQ: Autism Spectrum Quotient
Asp: Aspartate
BDI-II: Beck Depression Inventory
bHB: Beta-hydroxybutyrate
BMI: Body mass index
CSF: Cerebrospinal fluid
DNA: Deoxyribonucleic acid
DSM-5: Diagnostic and Statistical Manual of Mental Disorders 5th edition
EQ: Empathy quotient
FOV: Field of view
FVC: Fractional volume component
FWHM: Full width half maximum
GABA: Gamma-aminobutyric acid
GM: Gray matter
GPC: Glycerophosphocholine
GSH: Glutathione
Gln: Glutamine
Glu: Glutamate
ICD-10: International Classification of Diseases
mI: Myo-inositol
Lac: Lactate
Lac+: Lactate plus threonine
MD: Mitochondrial disease
MPRAGE: Magnetization prepared rapid gradient echo
MRI: Magnetic resonance imaging
MRS: Magnetic resonance spectroscopy
MWT-B: Multiple choice vocabulary test
NAA: *N*-acetylaspartate
NAAG: *N*-acetylaspartylglutamate
NTC: Neurotypical controls
PCC: Posterior cingulate cortex
PCh: Phosphocholine
PE: Phosphoethanolamine
RF: Radio frequency
SCL-90-R: Symptom-checklist-90®
sI: Scyllo-inositol
SNRI: Serotonin–norepinephrine reuptake inhibitor
SRS-2: Social Responsiveness Scale 2
SSRI: Selective serotonin reuptake inhibitors
STAI: State Anxiety Inventory
SVS: Single-voxel spectroscopy
Tau: Taurine
tCr: Total creatine
Thr: Threonine
TR: Repetition time
TE: Echo time
WM: White matter
WURS-k: Wender Utah Rating Scale
