# Supplementary material for: Increased cerebral lactate levels in adults with autism spectrum disorders compared to non-autistic controls: a magnetic resonance spectroscopy study
Source: Mol Autism. 2023 Nov 17;14:44. doi: 10.1186/s13229-023-00577-y (PMC10655272; doi:10.1186/s13229-023-00577-y)
Supplement: Supplementary file 1 — Additional file 1. Supplementary information, tables and figure. [file 13229_2023_577_MOESM1_ESM.docx]

**Supplementary materials**

**Supplementary Table 1: Consensus reporting standard****s for single-voxel 1H-MRS study according to Lin A et al., NMR in Biomedicine 2021.**

| 1. **Hardware** 2. Field strength: 3T 3. Manufacturer: Siemens 4. Model: PrismaFit 5. RF coil: 20 channels ^1^H- head coil 6. **Acquisition** 7. Pulse sequence: MEGA-semiLaser with and without water suppression 8. Volume of interest (VOI) locations: Posterior cingulate cortex 9. Nominal VOI size: 25 × 25 × 25 mm^3^ 10. Repetition time (T_R_) and echo time (T_E_): T_R_ = 1650 ms, T_E_ = 142 ms 11. Total number of acquisitions per spectrum: 384 averages (metabolites) and 16 for water reference 12. Spectral bandwidth: 1200 Hz; Number of spectral points: 1024 points 13. Water suppression method: water suppression enhanced through T1 effects (WET) (bandwidth = 60 Hz) 14. Shimming method: Automated B0-field mapping followed by manual shimming of water to <15 FWHM |
| --- |
| 1. **Analysis software and outputs** 2. Analysis software: Osprey v2.5.0 3. Standard processing steps. Except, fitting range: 0.5 to 3.5 ppm; baseline knot spacing: 0.55 ppm 4. Output measures: Combined absolute concentration of lactate and threonine (Lac+), in institutional units. 5. Quantification: linear-combination (Osprey) modeling (with robust spectral registration using statistical parametric mapping) and tissue correction and quantification is performed based upon modeling parameters and tissue segmentation |
| 1. **Fitting model basis set**   Osprey basis sets composed of: Ala, Asp, bHB, Cr, GABA, GPC, GSH, Gln, Glu, H2O, mI, Lac, NAA, NAAG, PCh, PE, sI, Tau, Thr, MM09, MM12, MM14, MM17, MM20, Lip09, Lip13, and Lip20 |
| 1. **Data quality** 2. Reported variables in Osprey: SNR, Full Width At Half Maximum (FHWM) 3. Data exclusion criteria: FHWM > 8 Hz, Cr signal to noise ratio (SNR) < 25, frequency shift < -12 Hz 4. Measures of SNR, FWHM (in ppm), and FreqShift, as reported by Osprey for included spectra: PCC (121 spectra), SNR [60.9 ± 12.9 (36.3 – 92.9)], FWHM [5.2 ± 0.48 (4.26 – 6.64) Hz], FreqShift [-7.43 ± 1.22 (-9.98 – -4.97)] 5. Sample spectrum: Figure 1 in supplementary |

**Supplementary Table 2: Group Differences in 1H-MRS Quality Measures**

Abbreviations: fGM = fractional grey matter, fWM = fractional white matter, fCSF = fractional cerebrospinal fluid, Cr SNR = signal-to-noise ratio of the creatine signal, Cr FWHM = full width at half maximum of the creatine signal, water FWHM = full width at half maximum of the water signal, freqShift = frequency shift, relResA = relative residuals of the off-spectrum, relResdiff = relative residuals of the diff-spectrum.

| **Characteristic** | **ASD**, N = 64^1^ | **NTC**, N = 58^1^ | **p-value**^2^ |
| --- | --- | --- | --- |
| fGM | 0.48 ± 0.08 | 0.50 ± 0.09 | 0.4 |
| fWM | 0.41 ± 0.08 | 0.41 ± 0.09 | 0.9 |
| fCSF | 0.10 ± 0.03 | 0.09 ± 0.03 | 0.065 |
| Cr SNR | 59.9 ± 12.7 | 62.1 ± 13.2 | 0.3 |
| Cr FWHM | 5.3 ± 0.5 | 5.1 ± 0.4 | 0.2 |
| Water FWHM | 5.6 ± 1.2 | 5.8 ± 0.8 | >0.9 |
| freqShift | -7.2 ± 1.1 | -7.7 ± 1.3 | 0.075 |
| relResA | 3.5 ± 6.6 | 2.7 ± 0.6 | 0.6 |
| relResdiff1 | 1.8 ± 1.4 | 1.7 ± 0.5 | 0.4 |
| ^1^Mean ± SD | | | |
| ^2^Wilcoxon rank sum test | | | |

**Supplementary Table 3: Analysis of the effect of the 'group' factor and other potential confounders on various Lac+ measurements, as well as tNAA and tCr.**

Abbreviations: DFn = between groups degrees of freedom, DFd = within groups degrees of freedom; FVC1 and FVC2 = fractional volume components derived from the voxel’s fractional grey matter, white matter, and cerebrospinal fluid using principal component analysis, freqShift = frequency shift, relResdiff = relative residuals of the diff-spectrum, tNAA = total N-acetylaspartate, tCr = total creatine, Lac+ = lactate plus threonine, CSF = cerebrospinal fluid.

| metabolite | Effect | DFn | DFd | F | p | p<.05 |
| --- | --- | --- | --- | --- | --- | --- |
| Non-adjusted Lac+ metabolite amplitude | group | 1 | 114 | 8.765 | 0.004 | * |
|  | FVC1 | 1 | 114 | 0.053 | 0.819 |  |
|  | FVC2 | 1 | 114 | 4.780 | 0.031 | * |
|  | relResdiff1 | 1 | 114 | 2.639 | 0.107 |  |
|  | freqShift | 1 | 114 | 3.272 | 0.073 |  |
|  | age | 1 | 114 | 13.599 | <0.001 | * |
|  | sex | 1 | 114 | 5.253 | 0.024 | * |
| water-scaled and CSF corrected Lac+ concentration | group | 1 | 114 | 4.559 | 0.035 | * |
|  | FVC1 | 1 | 114 | 4.653 | 0.033 | * |
|  | FVC2 | 1 | 114 | 1.982 | 0.162 |  |
|  | relResdiff1 | 1 | 114 | 1.978 | 0.162 |  |
|  | freqShift | 1 | 114 | 0.090 | 0.765 |  |
|  | age | 1 | 114 | 1.188 | 0.278 |  |
|  | sex | 1 | 114 | 0.490 | 0.485 |  |
| Raw water-scaled Lac+ concentration | group | 1 | 114 | 4.312 | 0.040 | * |
|  | FVC1 | 1 | 114 | 3.782 | 0.054 |  |
|  | FVC2 | 1 | 114 | 0.777 | 0.380 |  |
|  | relResdiff1 | 1 | 114 | 2.068 | 0.153 |  |
|  | freqShift | 1 | 114 | 0.071 | 0.790 |  |
|  | age | 1 | 114 | 1.150 | 0.286 |  |
|  | sex | 1 | 114 | 0.483 | 0.489 |  |
| Lac+/tCr | group | 1 | 114 | 5.092 | 0.026 | * |
|  | FVC1 | 1 | 114 | 8.173 | 0.005 | * |
|  | FVC2 | 1 | 114 | 13.408 | <0.001 | * |
|  | relResdiff1 | 1 | 114 | 0.336 | 0.563 |  |
|  | freqShift | 1 | 114 | 1.818 | 0.180 |  |
|  | age | 1 | 114 | 0.017 | 0.896 |  |
|  | sex | 1 | 114 | 2.990 | 0.086 |  |
| water-scaled and CSF corrected and tissues-corrected Lac+ concentration | group | 1 | 114 | 4.669 | 0.033 | * |
|  | FVC1 | 1 | 114 | 12.630 | 0.001 | * |
|  | FVC2 | 1 | 114 | 10.645 | 0.001 | * |
|  | relResdiff1 | 1 | 114 | 2.052 | 0.155 |  |
|  | freqShift | 1 | 114 | 0.077 | 0.781 |  |
|  | age | 1 | 114 | 1.183 | 0.279 |  |
|  | sex | 1 | 114 | 0.528 | 0.469 |  |
| tNAA tissue corrected | group | 1 | 114 | 1.546 | 0.216 |  |
|  | FVC1 | 1 | 114 | 27.458 | <0.001 | * |
|  | FVC2 | 1 | 114 | 5.406 | 0.022 | * |
|  | relResdiff1 | 1 | 114 | 1.460 | 0.229 |  |
|  | freqShift | 1 | 114 | 6.041 | 0.015 | * |
|  | age | 1 | 114 | 18.789 | <0.001 | * |
|  | sex | 1 | 114 | 2.585 | 0.111 |  |
| Log-transformed tCr tissue corrected | group | 1 | 114 | 0.056 | 0.813 |  |
|  | FVC1 | 1 | 114 | 162.920 | <0.001 | * |
|  | FVC2 | 1 | 114 | 0.640 | 0.426 |  |
|  | relResdiff1 | 1 | 114 | 0.954 | 0.331 |  |
|  | freqShift | 1 | 114 | 3.663 | 0.058 |  |
|  | age | 1 | 114 | 5.235 | 0.024 | * |
|  | sex | 1 | 114 | 4.338 | 0.040 | * |


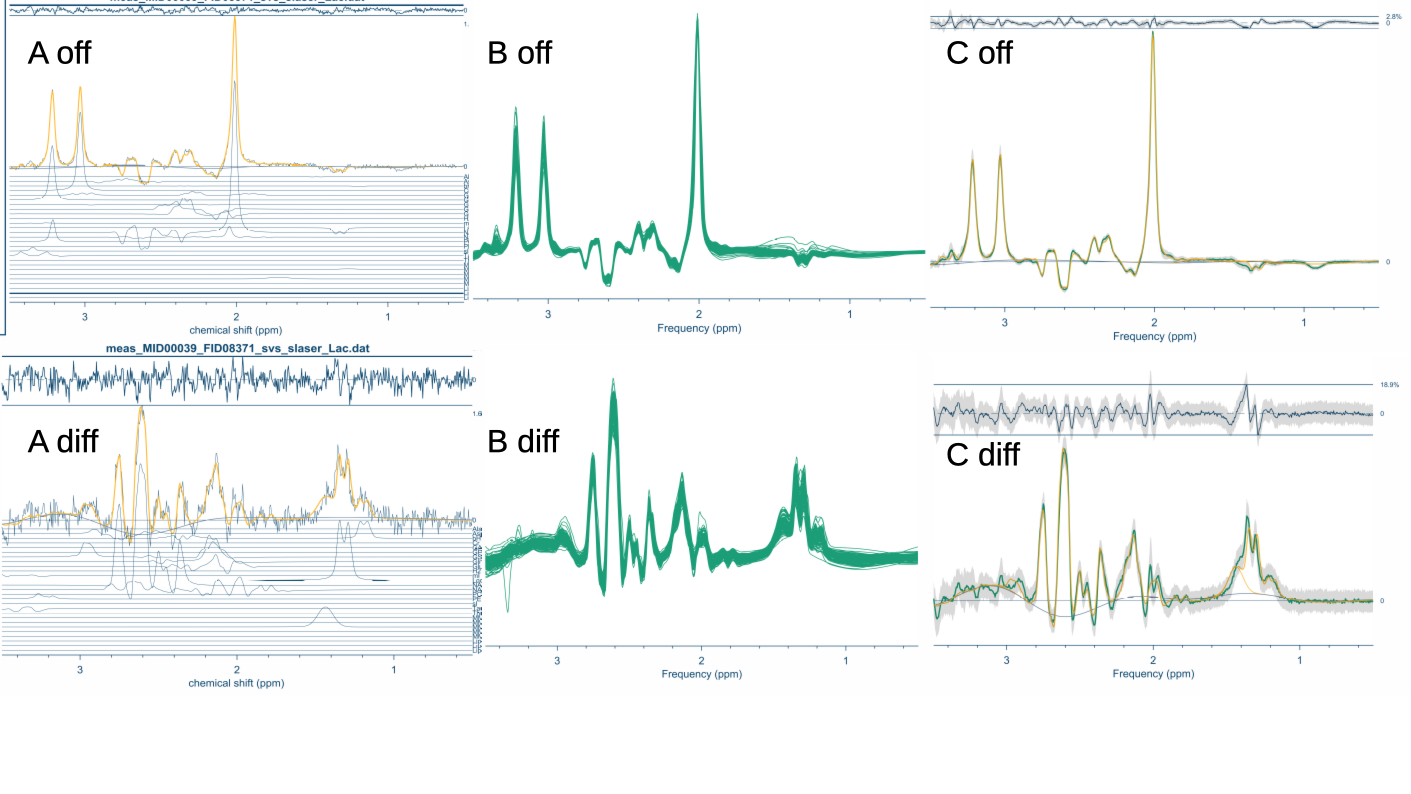


**Supplementary Figure 1.** Representative proton magnetic resonance spectra of the posterior cingulate cortex (PCC) processed and modeled via Osprey using MEGA-semiLaser data. (A) Exemplary spectral fit of the OFF and DIFF spectra of one participant with contributions from individual metabolites delineated. (B) Individual OFF and DIFF spectra showcasing variations. (C) Aggregated mean OFF and DIFF spectra depicted in green with standard deviation represented by gray shaded areas; overlay of mean spectral fit in yellow and mean residual shown above.

**Supplementary Information 1:**

To determine the most suitable volume of interest (VOI) for our study, we assessed signal quality in various brain regions by measuring the Full Width at Half Maximum (FWHM) of water peaks. Lower FWHM values indicate better signal quality. The regions we probed were selected based on their relevance to the cognitive functions under investigation. Specifically, the FWHM values obtained were:

- **Posterior Cingulate Cortex**: 13.3 Hz
- **Pregenual Anterior Cingulate Cortex**: 19.2 Hz
- **Dorsal Anterior Cingulate Cortex**: 14.9 Hz
- Medial Cingulate Cortex: 14.8 Hz
- **Dorsolateral Prefrontal Cortex**: 14.6 Hz

Among the areas tested, the Posterior Cingulate Cortex (PCC) exhibited the lowest FWHM value.

**Supplementary Information 2: Fractional volume effects**

Informed by theories such as the lactate-shuffle theory, suggesting differences in lactate concentrations across grey matter (GM), white matter (WM), and cerebrospinal fluid (CSF), we conducted an empirical investigation. We applied principal component analysis (PCA) on fractional GM, WM, and CSF volumes within a voxel, resulting in two components (FVC1 and FVC2) that accounted for all the variance (64% and 36% respectively). We then set up several ANCOVA models to examine the effect of fractional voxel volumes, with group, age, relative Residual Differences, FVC1, FVC2, sex, and frequency shift as independent variables. Five Osprey outputs were used as dependent variables. The aim was to determine which output best minimized the fractional volume effect. Our analyses indicated a significant effect of FVC1 and / or FVC2 on all outputs except raw water-scaled lactate concentration (see **Supplementary Table 3**). Osprey’s tissue correction introduced fractional volume effects for FVC1, likely due to the lack of established reference values for GM/WM lactate concentrations in the current literature needed for precise tissue correction. Therefore, raw water-scaled lactate concentration emerged as the most accurate measure to account for fractional volume differences and will be used as Lac+ in subsequent analyses.

**Supplementary Information 3: Confirmatory analyses regarding other Lac+ estimates derived from Osprey**

In supplementary analyses (cf. Supplementary Table 3), we examined the effect of the "group" factor on the other lactate measures (unadjusted Lac+ metabolite amplitudes, water-scaled and CSF-corrected Lac+ concentrations, water-scaled plus CSF-corrected plus tissue-corrected Lac+ concentrations, and the Lac+/tCr ratio) by ANCOVA models including covariates such as FVC1, FVC2, sex, age, and relative residuals to account for potential confounders, particularly the fractional volume effect.

After controlling for confounders, all other lactate estimates (including unadjusted lactate metabolite amplitudes, water-scaled and CSF-corrected lactate concentrations, water-scaled plus CSF-corrected plus tissue-corrected lactate concentrations, and Lac+/tCr ratio) showed a significant group effect. However, no significant group effect was found in the exploratory analyses of tNAA and log-transformed tCr.

**Supplementary Information 4: Within-Person variability of the Lac+ signal**

To evaluate the within-person variability of the Lac+ signal, we conducted a longitudinal assessment on one of the authors. Measurements were taken twice daily—once in the morning and once in the evening—over a period of two days. The lactate levels did show some fluctuation but remained within a relatively narrow range. Specifically, the lactate concentration was 2.42 IU in the morning and 2.22 IU in the evening on Day 1. On Day 2, the lactate concentration was 1.92 IU in the morning and 1.86 IU in the evening.
